# Anaphylactic shock in a patient with severe aortic stenosis treated with adrenaline and landiolol for circulatory management: A case report

**DOI:** 10.1097/MD.0000000000027135

**Published:** 2021-09-03

**Authors:** Akihiro Yokoyama, Motohiro Sekino, Taiga Ichinomiya, Hironori Ishizaki, Keiko Ogami-Takamura, Takashi Egashira, Rintaro Yano, Sojiro Matsumoto, Ushio Higashijima, Tetsuya Hara

**Affiliations:** a Department of Anesthesiology and Intensive Care Medicine, Nagasaki University Graduate School of Biomedical Sciences, Nagasaki, Japan; b Department of Macroscopic Anatomy, Nagasaki University Graduate School of Biomedical Sciences, Nagasaki, Japan; c Department of Intensive Care, Nagasaki Harbor Medical Center, Nagasaki, Japan.

**Keywords:** β1-selective antagonist, β-blockade, anaphylaxis, cardiac contractility, epinephrine, heart rate, landiolol, myocardial contractility

## Abstract

**Rationale:**

We present the first case of a patient with severe aortic stenosis who developed anaphylactic shock and was successfully treated with adrenaline and landiolol, a highly selective β1-receptor blocker, to prevent disruption of the myocardial oxygen supply–demand balance caused by tachycardia.

**Patient concerns:**

An 86-year-old woman was scheduled for simultaneous anterior–posterior fixation for a burst fracture of the 12th thoracic vertebra; 200 mg sugammadex, a neuromuscular blocking agent antagonist, was administered postoperatively, and she was extubated without complications. However, 6 min after extubation, her blood pressure decreased abruptly to 55/29 mm Hg, and her heart rate increased to 78 bpm. Then, we intervened with fluid loading, an increased dose of noradrenaline, and phenylephrine administration. However, her blood pressure did not increase.

**Diagnoses:**

A general observation revealed urticaria on the lower leg; thus, we suspected anaphylactic shock due to sugammadex administration.

**Interventions:**

We carefully administered 2 doses of 0.05 mg adrenaline and simultaneously administered landiolol at 60 μg/kg/min to suppress adrenaline-induced tachycardia. Adrenaline administration resulted in a rapid increase in blood pressure to 103/66 mm Hg and a maximum heart rate of 100 bpm, suppressing excessive tachycardia.

**Outcomes:**

The patient's general condition was stable after the intervention, and circulatory agonists could be discontinued the following day. She was discharged from the intensive care unit on the fourth postoperative day.

**Lessons:**

Landiolol may help control the heart rate of patients with aortic stenosis and anaphylactic shock. The combined use of landiolol and adrenaline may improve patient outcomes; however, their efficacy and risks must be evaluated by studying additional cases.

## 1 Introduction

Patients with aortic stenosis often develop compensatory left ventricular hypertrophy. Myocardial oxygen consumption increases due to left ventricular hypertrophy, resulting in an imbalance between myocardial oxygen supply and demand, leading to an increased risk of congestive heart failure and sudden death.^[1,2]^ To increase myocardial oxygen supply and maintain the myocardial oxygen supply–demand balance in patients with hypertrophic cardiomyopathy and aortic stenosis, it is crucial to maintain an appropriate heart rate, normal left ventricular diastolic volume (preload), and normal systemic vascular resistance (afterload), while limiting myocardial contractility and maintaining coronary blood flow.^[1,3]^

Anaphylaxis in patients with aortic stenosis induces an acute increase in heart rate and myocardial contractility while also reducing the preload and afterload. This may further lead to rapid deterioration in circulation. Additionally, the administration of adrenaline, a key drug to reverse anaphylaxis, may further increase heart rate and myocardial contractility, and these effects may disrupt the myocardial oxygen supply–demand balance, leading to cardiac arrest. There are no guidelines for treating anaphylactic shock in patients with aortic stenosis; however, it seems apparent that adrenaline should be administered with utmost caution so as not to induce excessive tachycardia and myocardial contractility. It is challenging to suppress myocardial contractility induced by adrenaline in patients with anaphylactic shock; however, the heart rate should be tightly controlled. Landiolol is a highly selective β1-receptor blocker (β1/β2 ratio of 255). It is a short-acting drug with a blood half-life of approximately 4 min and has excellent adjustability. In addition, it is less likely to cause hypotension due to its weak negative inotropic effect.^[4]^ Therefore, landiolol may help control heart rate with minimal effect on blood pressure in patients with aortic stenosis and shock.^[5]^

Herein, we present the first case of a patient with severe aortic stenosis who developed anaphylactic shock due to sugammadex administration, a neuromuscular blocking agent antagonist, and was successfully treated with adrenaline and landiolol administration.

## 2 Case report

**Figure 1:**
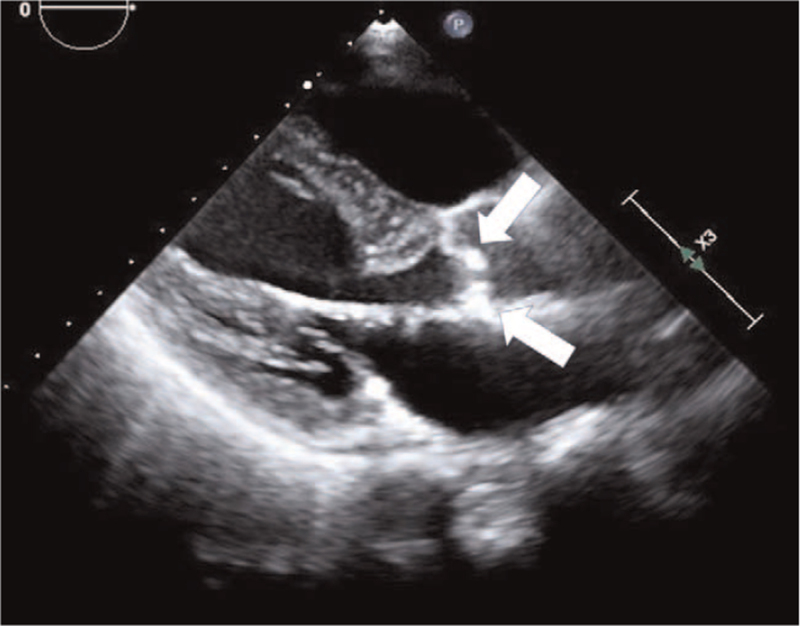
Transthoracic echocardiography findings. Transthoracic echocardiography long-axis view of the 4 cavities shows severe calcification of the aortic valve and restricted aortic valve leaflet motion during systole (white arrows).

**Figure 2:**
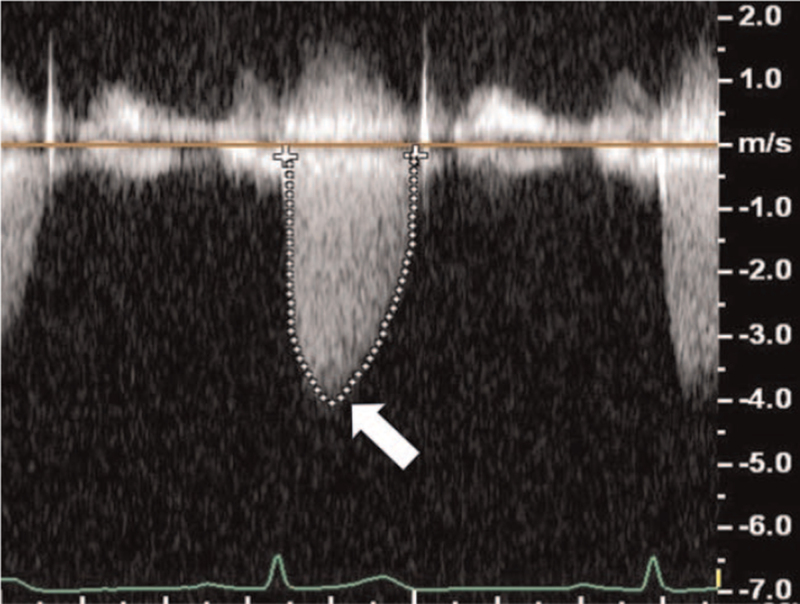
Doppler and tracing of the velocity curve of severe aortic stenosis jet. Doppler and tracing of the velocity curve of severe aortic stenosis jet showing a maximum blood flow velocity of 4.05 m/s (white arrow) and mean pressure gradient of 42 mm Hg.

An 86-year-old woman (weight, 45 kg; height, 148 cm; body mass index, 20.5 kg/m^2^) was scheduled for simultaneous anterior–posterior fixation for a burst fracture of the 12th thoracic vertebra. Her prescription medications included 1.25 mg bisoprolol (β1-selective β-blocker) and 5 mg amlodipine. The patient's medical history included severe aortic stenosis with a history of syncope. Preoperative echocardiography showed severe calcification of the aortic valve and restricted aortic valve leaflet motion during systole (Fig. 1), with a valve opening area of 0.72 cm^2^, maximum blood flow velocity of 4.05 m/s, and mean pressure gradient of 42 mm Hg (Fig. 2). Sevoflurane, remifentanil, and rocuronium were used for anesthesia; noradrenaline was continuously administered to maintain perfusion pressure after inserting a central venous catheter; intraoperative systolic blood pressure remained within the range of 100 to 120 mm Hg. Her heart rate remained within the range of 50 to 60 bpm. After completing the surgery, 200 mg sugammadex was administered, and the patient was extubated without complications. Following extubation, her blood pressure was 115/51 mm Hg, and her heart rate was 69 bpm. However, 6 min after extubation, her blood pressure decreased abruptly to 55/29 mm Hg, and her heart rate increased to 78 bpm. Then, we intervened with fluid loading, increased noradrenaline dosage from 0.03 to 0.3 μg/kg/min, and phenylephrine administration. However, her blood pressure did not increase. Transthoracic echocardiography showed no abnormalities, such as cardiac contractility. A general observation revealed urticaria on the lower leg; thus, we suspected anaphylactic shock due to sugammadex administration. Fortunately, there were no complaints of respiratory distress or wheezing, and oxygen saturation was 95% under a 5-L mask. At this point, her blood pressure was 56/30 mm Hg, and her heart rate was 85 bpm. Two doses of 0.05 mg adrenaline were intravenously administered for the treatment of anaphylactic shock. Simultaneously, to prepare for adrenaline-induced tachycardia, we initiated landiolol at 60 μg/kg/min while monitoring the response to adrenaline. Adrenaline administration resulted in a rapid increase in blood pressure to 103/66 mm Hg and a maximum heart rate of 100 bpm, thus suppressing excessive tachycardia. Additionally, continuous adrenaline infusion was initiated at 0.1 μg/kg/min to reduce circulatory variability, and landiolol was gradually reduced to achieve a target heart rate of <80 bpm. On admission to the intensive care unit (ICU), the patient's blood pressure was 120/52 mm Hg, and her heart rate was 91 bpm with 0.05 μg/kg/min adrenaline and 10 μg/kg/min landiolol. The patient's general condition was stable after admission to the ICU, and circulatory agonists could be discontinued the following day. We also administered 125 mg of methylprednisolone for 3 days with no antihistamines. She was discharged from the ICU on the fourth postoperative day. She was then transferred to a rehabilitation hospital on the 23rd postoperative day without any signs of exacerbating aortic stenosis or heart failure symptoms.

The patient provided written informed consent for the publication of this case report. This report was approved by the Institutional Review Board of Nagasaki University Hospital (Approval Number: 21051727).

## 3 Discussion

Herein, we present the case of a patient with severe aortic stenosis who developed anaphylactic shock and was successfully and safely treated with careful administration of adrenaline and landiolol, a highly selective β1-receptor β-blocker, to suppress adrenaline-induced tachycardia.

Adrenaline administration is an essential treatment for anaphylactic shock due to its strong α- and β-receptor-stimulating effects and an inhibitory effect on histamine release and other anaphylactic mediators.^[6,7]^ However, adrenaline may reduce coronary blood flow by inducing coronary vasoconstriction, increase myocardial oxygen consumption by increasing the heart rate and cardiac contractility due to the β1-receptor effect, and induce myocardial ischemia even in patients with anaphylactic shock who are originally at low risk of myocardial ischemia.^[8]^ Adrenaline administration in patients with aortic stenosis is even more likely to cause myocardial oxygen supply–demand imbalance.^[9]^ In patients with aortic stenosis or hypertrophic cardiomyopathy, improperly managed anaphylactic shock can lead to circulatory collapse and necessitate extracorporeal membrane oxygenation,^[10,11]^ although there are no definitive guidelines regarding the use of adrenaline in such patients. However, it seems apparent that adrenaline should be administered with utmost caution so as not to induce excessive tachycardia and myocardial contractility.

Highly selective β1-receptor blockers are candidates for treating excessive tachycardia induced by adrenaline administration in patients with anaphylactic shock. Propranolol, a nonselective β-receptor blocker, should not be administered because of the potential for increased severity of hypotension and anaphylaxis due to coronary vasospasm, increased vascular permeability, and chemical mediator release caused by the β2-receptor blockade.^[12,13]^ Highly selective β1-receptor blockers include landiolol and esmolol. The high β1 selectivity of landiolol (β1/β2 ratio of 255) is much better than the β1 selectivity of esmolol (β1/β2 ratio of 33)^[14,15]^. In addition, the blood half-life of randiolol is 4 min, which is ultrashort-acting compared to the 9-min blood half-life of esmolol. Therefore, it has excellent heart rate regulation^[14]^ and is very useful when strict heart rate control is required, as in the present case. Furthermore, it is superior to esmolol in that its negative inotropic effect is weaker than that of esmolol^[4,16]^. Therefore, it is less likely to cause hypotension and can control heart rate while minimizing the effect on blood pressure in patients with anaphylactic shock. Since landiolol can suppress the hemodynamic effects of adrenaline,^[17,18]^ it was administered in combination with the careful use of adrenaline to reduce myocardial oxygen consumption and maintain the myocardial oxygen supply–demand balance by suppressing tachycardia.

Patients taking β-blockers are less responsive to adrenaline during anaphylactic shock and are at an increased risk of anaphylactic shock.^[19]^ This is because the β-receptor-stimulating effect of adrenaline is not exerted, and the α-receptor-stimulating effect is dominant. Chemical mediators of anaphylaxis, such as histamine, are produced and released from mast cells and basophils and are inhibited by β2-receptor-stimulating effects; however, their release is enhanced when α-receptor-stimulating effects are dominant.^[20,21]^ As a result of α-receptor stimulation, peripheral blood vessels constrict, but a compensatory parasympathetic reflex occurs, resulting in bradycardia.^[22]^ Although the use of intravenous β-blockers at the onset of anaphylactic shock should be cautionary, highly selective β1 receptor-blockers have a weaker aggravating effect on anaphylactic shock, and adrenaline has a more substantial improving effect.^[12]^ The enhancement of the inhibitory effect on the release of chemical mediators by β2 receptors may have improved the symptoms of anaphylactic shock.^[20,21]^ Under strict monitoring of heart rate and blood pressure, we believe that highly selective β1-receptor blockers can be used effectively without aggravating anaphylactic shock.

To the best of our knowledge, this is the first case report documenting recovery from anaphylactic shock in a patient with severe aortic stenosis by the combined administration of adrenaline and landiolol. In anaphylactic shock patients with severe aortic stenosis who may experience circulatory collapse due to adrenaline-induced excessive tachycardia, landiolol may have a beneficial effect, thereby preventing myocardial ischemia and cardiac arrest. However, it is necessary to evaluate the efficacy and risk by accumulating cases and conducting sufficient case studies. Additionally, the most appropriate methods of administration and doses of adrenaline and landiolol are unknown. The recommended intravenous dose of adrenaline for treating perioperative anaphylactic shock is 0.05 mg^[23]^; however, adrenaline may affect the myocardial supply–demand balance in a dose-dependent manner, and its efficacy varies among individuals. In patients with myocardial ischemia caused by Kounis syndrome, it is proposed to start with the continuous administration of small doses and taper the dose while monitoring vital signs.^[24]^ Irrespective of intravenous or continuous intravenous administration of adrenaline, it is important to start with a small dose and taper the dose while monitoring the patient's condition and response to adrenaline. Landiolol should also be cautiously administered as it may cause a dose-dependent reduction in heart rate,^[25]^ and its efficacy varies among individuals. Especially, in patients taking β-blockers, as in the present case, landiolol should be used with caution because adrenaline's potent α-receptor-stimulating effect may cause severe bradycardia rather than tachycardia.^[22]^ Additionally, adrenaline may be ineffective in patients taking β-blockers; glucagon may be administered in this case.^[23]^ The administration of glucagon for refractory anaphylaxis in patients taking β-blockers has been described in several reports,^[26–28]^ although the level of evidence is low, and the recommended dose is 1 to 2 mg by intravenous administration.^[23]^ Glucagon exerts its effects by directly activating adenylyl cyclase in the myocardium, not via β-receptors,^[29]^ and may be useful in refractory anaphylaxis in patients taking β-blockers.

In the present case, the drug sugammadex was presumed to have caused anaphylaxis, but a diagnostic test, such as a skin test, did not confirm the diagnosis. None of the other administered drugs were suspected of having caused anaphylaxis. The frequency of sugammadex-induced anaphylaxis varies from 0.0029% to 0.039%, which is low.^[30,31]^

## 4 Conclusion

The management of anaphylactic shock in patients with severe aortic stenosis is dangerous and challenging. While avoiding excessive tachycardia, which induces rapid hemodynamic deterioration, we successfully and safely administered adrenaline and landiolol. The combined use of these drugs may have improved patient outcomes; however, the efficacy and risks need to be evaluated by accumulating cases.

## Acknowledgments

We would like to thank (www.editage.jp) for English language editing.

## Author contributions

**Conceptualization:** Akihiro Yokoyama, Motohiro Sekino, Taiga Ichinomiya, Hironori Ishizaki, Keiko Ogami-Takamura, Takashi Egashira, Rintaro Yano, Sojiro Matsumoto, Ushio Higashijima, Tetsuya Hara.

**Data curation:** Akihiro Yokoyama, Taiga Ichinomiya.

**Supervision:** Motohiro Sekino, Taiga Ichinomiya, Tetsuya Hara.

**Visualization:** Akihiro Yokoyama.

**Writing – original draft:** Akihiro Yokoyama, Motohiro Sekino.

**Writing – review & editing:** Akihiro Yokoyama, Motohiro Sekino, Taiga Ichinomiya, Hironori Ishizaki, Keiko Ogami-Takamura, Takashi Egashira, Rintaro Yano, Sojiro Matsumoto, Ushio Higashijima, Tetsuya Hara.
